# Learning to Detect Triggers of Airway Symptoms: The Role of Illness Beliefs, Conceptual Categories and Actual Experience with Allergic Symptoms

**DOI:** 10.3389/fpsyg.2017.00926

**Published:** 2017-06-07

**Authors:** Thomas Janssens, Eva Caris, Ilse Van Diest, Omer Van den Bergh

**Affiliations:** Health Psychology, KU LeuvenLeuven, Belgium

**Keywords:** asthma triggers, contingency learning, generalization (psychology), expectancy violation, illness perceptions

## Abstract

**Background:** In asthma and allergic rhinitis, beliefs about what triggers allergic reactions often do not match objective allergy tests. This may be due to insensitivity for expectancy violations as a result of holding trigger beliefs based on conceptual relationships among triggers. In this laboratory experiment, we aimed to investigate how pre-existing beliefs and conceptual relationships among triggers interact with actual experience when learning differential symptom expectations.

**Methods:** Healthy participants (*N* = 48) received information that allergic reactions were a result of specific sensitivities versus general allergic vulnerability. Next, they performed a trigger learning task using a differential conditioning paradigm: brief inhalation of CO_2_ enriched air was used to induce symptoms, while participants were led to believe that the symptoms came about as a result of inhaled allergens (conditioned stimuli, CS’s; CS+ followed by symptoms, CS- not followed by symptoms). CS+ and CS- stimuli either shared (e.g., birds-mammals) or did not share (e.g. birds-fungi) category membership. During Acquisition, participants reported symptom expectancy and symptom intensity for all triggers. During a Test 1 day later, participants rated symptom expectancies for old CS+/CS- triggers, for novel triggers within categories, and for exemplars of novel trigger categories. Data were analyzed using multilevel models.

**Findings:** Only a subgroup of participants (*n* = 22) showed differences between CO_2_ and room air symptoms. In this group of responders, analysis of symptom expectancies during acquisition did not result in significant differential symptom CS+/CS- acquisition. A retention test 1 day later showed differential CS+/CS- symptom expectancies: When CS categories did not share category membership, specific sensitivity beliefs improved retention of CS+/CS- differentiation. However, when CS categories shared category membership, general vulnerability beliefs improved retention of CS+/CS- differentiation. Furthermore, participants showed some selectivity in generalization of symptom expectancies to novel categories, as symptom expectancies did not generalize to novel categories that were unrelated to CS+ or CS- categories. Generalization to novel categories was not affected by information about general vulnerability or specific sensitivities.

**Discussion:** Pre-existing vulnerability beliefs and conceptual relationships between trigger categories influence differential symptom expectancies to allergic triggers.

## Introduction

Asthma and allergic rhinitis are chronic conditions that are characterized by an allergic or hyperreactive response of the airways to a variety of triggers (Bousquet et al., 2012; Global Initiative for Asthma (GINA), 2016). Because treatment for these conditions is currently not available, management strategies are suggested to reduce the manifestation of symptoms and increase clinical control (Global Initiative for Asthma (GINA), 2016). These management strategies are multifaceted, and include pharmacological strategies (often a combination of preventer and reliever medication) as well as behavioral strategies of trigger identification and subsequent avoidance as a way to obtain control over symptoms (Global Initiative for Asthma (GINA), 2016). However, despite these treatment options day-to-day control over symptoms is often poor (Rabe et al., 2004; Peters et al., 2007).

One reason for the lack of day-to-day symptom control may be the difficulties that arise when implementing trigger identification and behavioral avoidance strategies (Janssens and Ritz, 2013). These latter strategies rely on the perception of spatio-temporal contingencies between the presence of triggers and subsequent emergence of asthmatic or allergic symptoms in order to allow prediction of symptoms and accurate avoidance of triggers. In other words, based on medical information and personal experiences, patients construct trigger beliefs to guide their (future) behavior. Interestingly, trigger beliefs often do not match with the results of a structured trigger evaluation procedure, with both false positives and false negatives being observed (Li et al., 2000; Smith et al., 2009). Furthermore, in day-to-day asthma management, individuals with asthma often report being uncertain about their personal triggers and trigger avoidance strategies (Caress et al., 2002; Trollvik and Severinsson, 2004). In addition, individuals show a marked variation in the type and number of asthma triggers they identify, with a higher number of self-identified asthma triggers being associated with worse asthma outcomes, even when controlling for other measures of asthma severity (Ritz et al., 2006, 2016; Janssens and Harver, 2015). Taken together, these findings suggest difficulties and inaccuracies in the process of asthma trigger identification or the detection of trigger-symptom contingencies. Moreover, literature on symptom perception suggests that these beliefs about trigger-symptom contingencies may in turn bias perception of respiratory symptoms (Janssens et al., 2009; von Leupoldt and Dahme, 2012), which may lead to even more difficulties in trigger identification.

Previously, we have highlighted similarities between asthma trigger learning and other contingency learning tasks that occur in a motivational context, such as the identification of danger and safety that occurs within the context of fear learning (Janssens and Ritz, 2013; Janssens et al., 2015). Building upon these similarities, we have explored generalization of symptom-trigger contingencies as a potential mechanism of the observed inaccuracies in asthma trigger identification. Similar to conceptualization of generalization in the context of anxiety and fear, generalization of trigger beliefs may serve an adaptive purpose in that it helps to transfer knowledge that is gained from experience to similar instances which have not (yet) been experienced, therefore limiting the risk of adverse symptom outcomes. However, generalization may also be considered excessive or maladaptive when innocuous stimuli are treated as threatening, especially if the associated symptoms and behavioral responses interfere with day to day functioning or quality of life (Dunsmoor et al., 2009; van Meurs et al., 2014). An illustrative example in the field of allergy is the avoidance of tree nuts by individuals that show a sensitivity to peanut allergens. This avoidance seems sensible, based on considerable similarities between peanuts and tree nuts. However, a recent review of the available evidence for this strategy shows that avoidance of all tree nuts in individuals with peanut allergy may be overly precautious (Brough et al., 2015).

So far, in associative learning research, most research on generalization has studied perceptual similarities as a basis for generalization. However, recent research has also explored the role of higher order cognitions such as category membership and stimulus typicality as a basis for generalization, showing that participants can use their pre-existing knowledge about categories as a basis for fear generalization (Dunsmoor and Murphy, 2015; Dymond et al., 2015). Based on these developments in fear generalization research, we previously have adapted an associative learning or conditioning paradigm focusing on category based fear learning (Dunsmoor et al., 2012) into a lab method to investigate category-based respiratory trigger learning. Briefly, this method consists of the presentation of pictures, which are unique exemplars of two different allergen categories (e.g., mammals and flowers). Exemplars of one category (conditioned stimuli, CS+) predict onset of respiratory symptoms, whereas exemplars of the other category (CS-) are never followed by symptoms. Using this method, we observed generalization of trigger beliefs to novel category exemplars, as well as to exemplars of categories that were similar of the original trigger categories, providing a proof of concept that trigger beliefs are shaped by pre-existing conceptual knowledge (Janssens et al., 2015). Moreover, an important finding of this study was that generalization of symptom expectancies to novel CS+ exemplars was increased if participants had experienced CS+ and CS- categories that were more similar (e.g., mammals and birds), compared to categories that were more different (e.g., mammals and molds). We interpreted this finding as an effect of discrimination learning on the inferred relevance of category features as basis for generalization, which is in line with other studies that have showed an impact of either inferred or instructed feature relevance on feature based fear generalization, and support feature-extraction or rule-based accounts of generalization (Vervliet et al., 2010; Vervliet and Geens, 2014; Ahmed and Lovibond, 2015a,b).

The role of category identification and feature extraction in the generalization of cue-outcome contingencies prompts investigation into the potential role of other complex cognitive mechanisms in changing the course of generalization. More specifically, it may provide opportunities to link research on generalization with the large body of research on the role of illness-related beliefs in the context of symptom perception and disease-related behaviors (Leventhal et al., 1980; Hagger and Orbell, 2003). In asthma, research within this framework has been successful in highlighting the role of beliefs about symptom chronicity, controllability, and medication necessity and concerns, in explaining individual differences in symptom perception and medication use patterns (Horne and Weinman, 1999; Halm et al., 2006; Kaptein et al., 2010). However, research into beliefs about causality and beliefs about trigger-symptom causal chains have been limited. An exception to this is a study by McQuaid et al. (2002), who studied the cognitive complexity of causal understanding in children with asthma and their parents. In this study, participants were asked to elaborate on the question “what causes your asthma,” and “how does this trigger cause asthma symptoms.” Results of this study showed a variety of complexity of responses, ranging from phenomism (no differentiation between cause and effect) to complex psychophysiological causal models, with more complex understanding of causal chains in asthma being associated with better treatment strategies.

Building upon this study, the aim of our research was to investigate the relationship between beliefs about causality in asthma and the way individuals integrate real life experiences into models of symptom-trigger contingency. Our study provides a lab based analog for a common task in the initial treatment phase of allergy management: individuals receive information about what asthma is, and are confronted with a variety of potential triggers, that are linked to adverse outcomes (airway symptoms) in a probabilistic way. In line with our focus on causality, we chose to focus on beliefs that link asthma triggers to a general vulnerability vs. beliefs that focus on asthma triggers as very specific indicators of specific airway sensitivities, thereby mimicking different information that may be given to patients with allergic conditions by their physician or information individuals may find on the internet (Smith et al., 1998; Croft and Peterson, 2002; Huckvale et al., 2012). The actual contingencies that were presented in the task did not fully confirm or disconfirm this prior information, in that during acquisition, each potential trigger that was presented was unique. However, participants could use their knowledge of category membership and category relations to infer differences in trigger-symptom contingencies at a category level. We hypothesized that a focus on general vulnerability would hinder differentiation between triggers and non-triggers, whereas a focus on specific sensitivities would improve differentiation between triggers and non-triggers. Furthermore, in line with our previous findings on differential acquisition of trigger beliefs, we expected that the use of CS- trigger categories that were more similar to the CS+ categories would enhance differentiation between CS+ and CS- trigger beliefs.

## Materials and Methods

### Participants

The study was approved by the Social and Societal Ethics Committee at KU Leuven and the Ethical Review Board of Leuven University Hospitals (study ID: ML10101). Participants were 48 healthy volunteers (15 male, aged 17–38), recruited from the student population. Psychology students received course credit for participation in the experiment. The other participants received 12 euros.

Exclusion criteria were self-reported allergies, hay fever, asthma or other lung disease, heart disease, epilepsy, other severe medical or psychiatric illnesses and the presence of electronic implants. Furthermore, participants were excluded if their lung function (forced expiratory volume in 1 s) was below 80% of their predicted value.

### Materials

#### Measures

Symptom expectancy was measured using a visual analog scale (VAS) anchored at *definitely no symptoms* and *definite symptoms*. For symptom intensity and unpleasantness, VAS were used with the anchors *not at all intense/unpleasant* and *maximal imaginable intensity/unpleasantness*.

During the online retention/generalization test, for all pictures in the trigger stimulus set, participants rated whether they had seen the picture during the lab task, or whether it was novel. Furthermore, symptom probabilities were assessed on an 11-point scale ranging from 0% (will not experience symptoms), to 100% (will definitely experience symptoms).

The Positive and Negative Affect Schedule [PANAS; (Watson et al., 1988), Dutch version (Engelen et al., 2006)] was used to assess trait positive affect and trait negative affect. The PANAS is a 20 item scale consisting of positive and negative emotion words. For each of the items, participants indicate on a 5-point scale, ranging from *very little* to *very much*, to which extend they experience each of these feelings in their daily lives.

Suffocation fear was measured using the suffocation scale of the Dutch Claustrophobia Questionnaire (CLQ; Van Diest et al., 2010). This scale consists of 14 situations that may elicit suffocation fears. Participants rate how fearful they would feel in each of the situations, on a 5-point scale ranging from *not at all fearful* to *extremely fearful*.

#### Stimuli

*Asthma trigger stimuli* consisted of four categories of potential asthma triggers: mammals, birds, flowers, and molds. This is the same stimulus set that we have used in previous research (Janssens et al., 2015). Each category consists of 20 unique pictures, and stimulus categories can be organized into two hierarchical categories: “animals” (mammals; birds) and “plants”(flowers; molds), creating the potential for constructing acquisition trigger sets with CS’s that are conceptually more/less similar. The difference in similarity between category pairs was tested and confirmed in previous research (Janssens et al., 2015). Allocation of CS+/- categories during acquisition was counterbalanced across participants, according to **Table 1**.

**Table 1:** Trigger stimulus set and category relations.

CS+ and CS- conceptually similar			
**CS+**	**CS**-	**Gu**	
Flowers	Molds	Birds, Mammals	
Molds	Flowers	Birds, Mammals	
Birds	Mammals	Flowers, Molds	
Mammals	Birds	Flowers, Molds	

**CS+ and CS- conceptually dissimilar**			
**CS+**	**CS**-	**G+**	**G**-
Flowers	Birds	Molds	Mammals
Molds	Mammals	Flowers	Birds
Birds	Molds	Mammals	Flowers
Mammals	Flowers	Birds	Molds

*G+, generalization stimuli conceptually related with CS+; G-, generalization stimuli conceptually related with CS; Gu, generalization stimuli unrelated to CS+ or CS-.*

*Asthma information* was embedded in the informed consent form. In the general vulnerability condition, this consisted of information that asthma was an allergic condition, and that allergic responses to allergy triggers were an indication of a general vulnerability making it necessary to avoid all potential asthma triggers. The condition highlighting specific sensitivities consisted of information that asthma was the result of an allergic response to specific allergens, and that careful investigation of triggers and non-triggers was possible, so that individuals with asthma do not need to avoid a variety of potential triggers.

#### Apparatus

Lung function was measured using a spirometer (Jaeger Masterscope; Hoechberg, Germany) prior to the actual start of the experimental breathing trials. For the latter trials, a valve was used for switching between the regular room air and the CO_2_-enriched air. The CO_2_-enriched air consisted of a mixture of 7.5% CO_2_, 21% O_2_, and 71.5% N_2_ fed into a meteorological balloon. Short-term inhalation of CO_2_-enriched air affects respiration, increasing breathing frequency and volume, and feelings of breathlessness, mimicking aspects of asthma symptoms (De Peuter et al., 2008; Janssens et al., 2011). The participants breathed into a mask connected to the valve through an antibacterial filter. The mask was also connected to a capnograph (Nonin LifeSense, Leek, The Netherlands) and a pneumotachograph (Fleisch No. 2, fg-deutschland; Hechingen, Germany). Affect 4.0 software (Spruyt et al., 2010) was used for stimulus presentation and to record participant responses and capnograph and pneumotachograph signals.

### Procedure

When participants arrived at the laboratory, they received oral and written information about the experiment. Participants were told that they would inhale a series of aerosols, each containing a mixture of air and a specific allergen, and that there was a risk of the occurrence of respiratory symptoms during these breathing trials. The information about the experiment also included our asthma information manipulation, and participants were randomly assigned to receive information focusing on general vulnerability or specific sensitivities.

After reviewing the information and exclusion criteria, participants completed informed consent. Subsequently, lung function was measured.

Subsequently, trigger acquisition trials started using a similar trial-unique acquisition procedure as in Janssens et al. (2015). The experimenter left the room and participants received 20 breathing trials. Each breathing trial followed the same pattern. First, a novel picture of a potential asthma trigger was shown, indicating to the participant that this allergen would be presented during the breathing trial (although in reality no allergens were present and symptom onset and trigger-symptom contingency was experimentally controlled). Ten pictures randomly chosen from each the CS+ and CS- trigger category was used for this purpose. After presentation of the picture, participants rated symptom expectancy using the VAS expectancy scale. Subsequently, participants were instructed to breathe through the mask, while the picture remained visible. Through the mask, the participants inhaled either regular room air either CO_2_-enriched air. For 6 out of the 10 CS+ trials, participants inhaled CO_2_-enriched air followed after the pictures. In all other trials, participants inhaled room air. After 60 s, participants could take off the mask, and rated symptom intensity and unpleasantness, using the intensity and unpleasantness VAS scales. Ratings were followed by a 2-min recovery phase, after which participants were prompted to start a new breathing trial.

One day after trigger acquisition, participants filled out an online survey. The survey consisted of the PANAS and the Suffocation scale of the CLQ, as well as recognition and symptom probability ratings of the full trigger picture set. Trigger pictures were presented in random order. After completion of the survey, participants were debriefed.

### Data Reduction and Data Analysis

In order to obtain data about breathing behavior, pneumotachograph and capnograph data were processed oﬄine using PSPHA (De Clerck et al., 2006), which resulted in breath-by-breath information of respiratory timing, respiratory volume, and fraction of end-tidal CO_2_ (FetCO_2_). Results of these analyses were further averaged for each acquisition trial.

Data analysis was carried out using SPSS 22 (IBM, Armonk, NY, United States). Symptom response to CO_2_ was defined as a significant within-person difference between symptom ratings after the CO_2_ trials, compared to room air trials, calculated using independent samples *t*-tests. If participants showed a *p* < 0.05 significant difference in symptom levels either on the symptom intensity or symptom unpleasantness ratings, they were deemed CO_2_ responders (*n* = 22). Participants not showing a significant difference were deemed non-responders (*n* = 26). Responders and non-responders did not differ in gender [*X^2^*(1) = 0.01, *p* = 0.938], age [*t*(46) = -0.17, *p* = 0.864], negative affectivity [*t*(46) = -0.71, *p* = 0.479], positive affectivity [*t*(46) = 0.09, *p* = 0.928] and fear of suffocation [*t*(46) = -0.96, *p* = 0.340], nor did they differ in assignment to information manipulation groups [*X*^2^(1) = 0.00, *p* = 1.000], or assignment of similar/different CS categories during acquisition [*X*^2^(1) = 0.34, *p* = 0.562]. Additionally, we explored difference in respiratory parameters of CO_2_ responders vs. non-responders. One participant was excluded from these analyses because of equipment failure. In a series of one-way repeated measures ANOVA’s, we found significant differences in the two groups for expiratory and inspiratory volume, minute ventilation, inspiratory drive, and FetCO_2_ and room air and the minute ventilation (**Table 2**).

**Table 2:** Differences in Symptom Report and Respiratory Parameters between CO_2_ Responders and Non-responders.

Respiratory	CO_2_ Responders		CO_2_ Non-responders			
Parameter	Mean	*SD*	Mean	*SD*	*t*	^∗^	*P*
Symptom intensity CO_2_ (VAS)	14.765	13.506	6.167	8.657	2.67	0.011
Symptom intenity RA (VAS)	2.958	4.810	3.731	5.614	–0.51	0.615
Symptom unpleasantness CO_2_ (VAS)	14.530	15.830	5.923	8.488	2.40	0.021
Symptom unpleasantness RA (VAS)	2.529	4.517	3.310	5.590	–0.526	0.602
Expiratory volume CO_2_ (1)	0.895	0.326	0.540	0.360	3.51	0.001
Expiratory volume RA (1)	0.789	0.320	0.545	0.392	2.30	0.026
Inspiratory volume CO_2_ (1)	0.859	0.340	0.506	0.298	3.79	<0.001
Inspiratory volume RA (1)	0.752	0.328	0.489	0.275	2.99	0.005
Inspiratory flow CO_2_ (1/s)	0.415	0.149	0.282	0.150	3.03	0.004
Inspiratory flow RA (1/s)	0.368	0.131	0.272	0.146	2.34	0.024
Fraction of end-tidal CO_2_ (%)	11.22	1.30	9.22	1.83	4.22	<0.001
Fraction of end-tidal RA (%)	8.95	1.02	7.90	1.34	2.93	0.005
Minute ventilation CO_2_ (1/min)	10.739	3.839	7.270	4.048	2.99	0.005
Minute ventilation RA (1/min)	9.407	3.368	6.981	4.068	2.19	0.035

*SD, standard deviation; RA, room air. ^∗^ df = 46 for VAS ratings, df = 45 for respiratory parameters.*

Using only the responder data, acquisition, retention, and generalization of trigger beliefs was evaluated using multilevel (linear mixed models) analysis. Multilevel models were chosen because these models are less restrictive in variance-covariance assumptions for repeated measures data compared to repeated measures ANOVA, are robust to unbalanced designs, and are less restrictive in the need of having fully nested or fully crossed designs (e.g., clear separation between- and within-subject effects) compared to (repeated measures) ANOVA (Cnaan et al., 1997). Therefore, these models provide an option to deal with the peculiarities of our trigger recognition/generalization dataset (e.g., all participants having CS+ and CS- trials, but having either 20 Gu trials or 10 G+ and 10 G- trials). Models were fitted using random intercepts to account for the data being nested within participants, and were estimated using Maximum Likelihood estimation. SPSS uses Satterthwaite approximation to determine df for *F*-tests/*t*-tests. Model fit was evaluated using Akaike’s Information Criterion (AIC). We carried out additional analyses on the full set of participants, while including CO2 responder status as an additional factor. For the CO2 responders, this did not result in major changes to our findings for retention and generalization of trigger beliefs. Results of these analyses are reported as Supplementary Material.

## Results

### Acquisition of Trigger Beliefs

**FIGURE 1:**
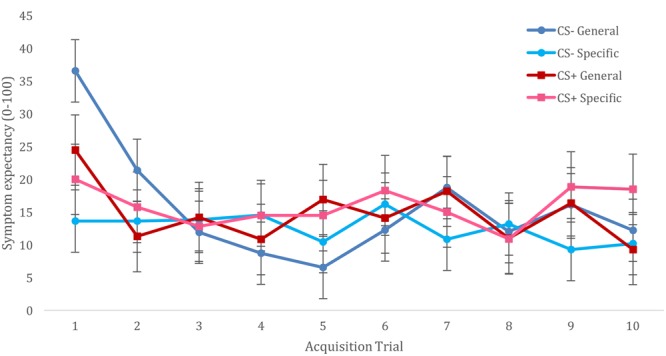
Trigger-symptom expectancies during acquisition phase.

For acquisition of trigger beliefs, we constructed a multilevel model that included fixed effects of CS (CS+ vs. CS-), Trial (T1–T10), and Trigger Information (general vulnerability vs. specific sensitivities), and included all interactions between these variables. The model also included a random (individual level) effect of CS, with an unstructured variance-covariance matrix. We observed no main effects of CS type [*F*(1,22) = 0.260, *p* = 0.615] nor a CS type × trial interaction [*F*(9,396) = 1.190, *p* = 0.300]. However, this analysis resulted in a significant main effect of trial [*F*(9,396) = 5.064, *p* < 0.001], showing reducing symptom expectancies from the first trial to subsequent trials. This effect was further qualified by Trigger Information [*F*(9,396) = 3.066, *p* = 0.001], showing that this decline in symptom expectancies was specific for participants who had been informed of triggers indicating general vulnerability. The CS × Information interaction was not significant [*F*(1,22) = 0.88, *p* = 0.358], and although the CS × Trial × Information interaction did not reach significance [*F*(9,396) = 1.692, *p* = 0.089], visual inspection of this interaction suggested better differentiation for CS+/CS- symptom expectancies when participants had been given information about triggers as specific sensitivities vs. general vulnerability, (cf. **Figure 1**). Addition of CS category relationship to these analyses did not result in improved model fit (AIC increased from 4504 to 3552) or changes in observed significant effects.

### Retention of Trigger Beliefs and Generalization to Novel Exemplars

**FIGURE 2:**
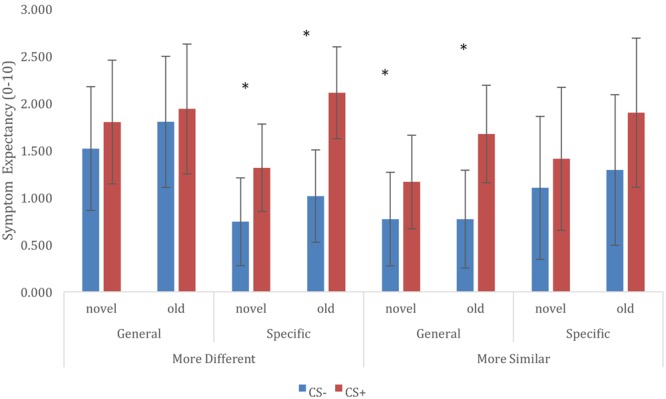
Retention of trigger-symptom expectancies, and generalization to novel trigger exemplars, depending on conditioned stimuli (CS), Trigger Information, and CS Category Relationship. ^∗^ CS+/CS– symptom expectancy differ at *p* < 0.05.

For retention of trigger beliefs, we constructed a multilevel model that included fixed effects of CS (CS+ vs. CS-), CS novelty (old vs. new), Category Relationship (similar vs. different), and Trigger Information (general vulnerability vs. specific sensitivities), and included all interactions between these variables. The model also included a random (individual level) intercept, to account for the data being nested within participants. Results of this analysis is represented in **Figure 2**. In general, Symptom expectancy was greater for CS+ compared to CS- exemplars [*F*(1,858) = 29.094, *p* < 0.001]. Furthermore, symptom expectancy was greater for old compared to novel trigger exemplars [main effect of CS novelty: *F*(1,858) = 11.231, *p* = 0.001]. This effect was unmodulated by interactions with any of the other model factors, and we observed differential symptom expectancies both for old [*t*(858) = 3.812, *p* < 0.001] as well for novel [*t*(858) = 3.995, *p* < 0.001] category exemplars. Finally, we observed a significant CS × Category Relationship × Trigger Information 3-way interaction [*F*(1,858) = 4.174, *p* = 0.041]. Further exploration of this interaction showed significant differential CS+/CS- expectancies when information was given about general vulnerability and CS categories were more similar [*t*(858) = 4.075, *p* < 0.001] or when information about specific sensitivities was given and CS categories were more different [*t*(858) = 5.409, *p* < 0.001], differences between CS+/CS- for other combinations of Trigger Information and CS Category Relationship were non-significant (but in the expected direction, cf. **Figure 2**). We did not observe any other significant main effects or interactions in this analysis.

### Generalization to Novel Trigger Categories

Based on the trigger categories that were used as CS+ and CS-, the novel trigger categories could be coded as G+ (related to CS+), G- (related to CS-) or Gu (unrelated to both CS categories). We constructed a multilevel model that included fixed effects of Stimulus Category (CS+, CS-, G+, G-, Gu), and Trigger Information (general vulnerability vs. specific sensitivities), and included all interactions between these variables. The model also included a random (individual level) intercept, to account for the data being nested within participants. Because of overlap of CS similarity with G categories (cf. **Table 1**), CS similarity was not added as a predictor to this model.

**FIGURE 3:**
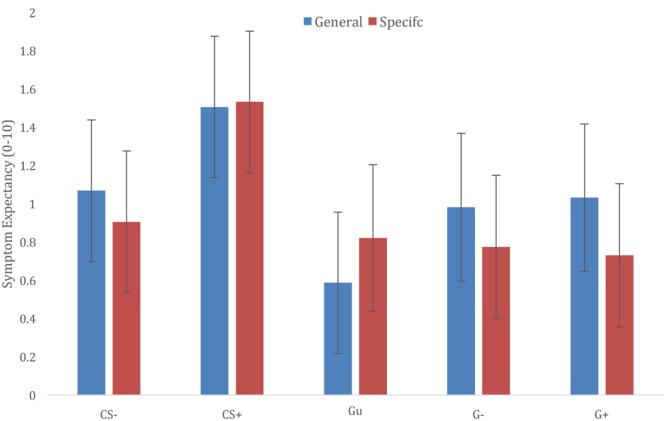
Generalization of trigger-symptom contingencies according to different information groups (general vulnerability vs. specific sensitivities).

Results showed a main effect of Stimulus Category [*F*(4,1745) = 34.832, *p* < 0.001], further exploration of this effect showed that CS+ ratings were significantly higher compared to all other categories [CS+/CS-*t*(1738) = 7.824, *p* < 0.001; CS+/Gu *t*(1751) = 9.588, *p* < 0.001; CS+/G-*t*(1746) = 7.044, *p* < 0.001; CS+/G+ *t*(1746) = 7.011, *p* < 0.001], and that symptom expectancies for CS- exemplars were higher compared to Gu symptom expectancies [*t*(1751) = 3.329, *p* = 0.009], but not different from G+ and G- symptoms expectancies [CS-/G+ *t*(1746) = 1.165, *p* > 0.99; CS-/G-*t*(1746) = 1.198, *p* > 0.99]. Gu, G+, and G- ratings did not differ from each other (cf. **Figure 3**).

The main effect of Trigger Information was not significant [*F*(1,22) = 0.025, *p* = 0.875], nor did the Trigger Information × Stimulus Category interaction yield a significant effect [*F*(4,1745) = 1.951, *p* = 0.100]. Visual exploration of the interaction suggested that providing information about specific sensitivities may prevent generalization to generalization categories that were related to CS categories (G+; G-), but not Gu category triggers (cf. **Figure 3**).

## Discussion

In this experiment, we used a laboratory analog task in order to investigate the impact of information about the causal structure of asthma triggers and symptoms (asthma triggers being an indication of general vulnerability vs. specific sensitivities) on the acquisition, retention, and generalization of category-based trigger-symptom contingencies.

Results of the acquisition phase did not show clear evidence for the acquisition of category based symptom expectancies. This lack of clear acquisition effects is contrary to previous results with a similar experimental method, in a study that did not include explicit information about general vulnerability or specific sensitivities (Janssens et al., 2015). This may suggest that both types of information hinder the acquisition of differential trigger expectancies, although the large number of non-responders in the current experiment may limit the value of this comparison (cf. supra).

During the retention and generalization phase, we did observe retention of category based symptom expectancies, and generalization of these expectancies to novel CS+/CS- category exemplars. Information about specific sensitivities or general vulnerability had an impact on symptom expectancies, but this effect was not straightforward, as it was moderated by the trigger category relationship. Information about specific sensitivities led to better retention of differential expectancies when CS Categories had been more different, whereas information about general vulnerability led to better retention when CS Categories had been more similar. At first sight, the emergence of differential symptom expectancies after the acquisition phase may be puzzling. However, it is possible that the abstraction of category level information from the unique exemplars does not happen right away, and therefore would not show up on the trial by trial expectancy ratings. Furthermore, previous studies have shown category level consolidation effects, extending to other CS+ exemplars (Dunsmoor et al., 2015), which could explain why we do find differential CS+/CS- retention effects in absence clear differential learning during acquisition.

When confronted with novel (generalization) trigger categories, we could not confirm our hypothesis that trigger expectancies generalize to trigger categories that are related to the CS categories. However, participants did show some selectivity in generalization to novel categories, as evidenced by our finding that symptom expectancies for Gu triggers were lower than expectancies for CS+ and CS- triggers. Interestingly, we did not observe any differences between G+ and G- category exemplars, although the limited number of participants precludes us from making strong inferences about this. Generalization to novel categories was not moderated by our information manipulation, although visual inspection of the results was in line with information about asthma being caused by a general vulnerability leading to stronger symptom expectancies for trigger categories that were similar to CS+ or CS- categories, but not to potential triggers from unrelated categories.

Despite the many interaction effects that we observed, the results in the different conditions of our experiment demonstrate the impact of prior information on the acquisition, retention, and generalization of category-based trigger-symptom contingency beliefs. As the information conditions in our experiment mimic aspects of trigger-related information or advice that is given to patients by physicians or in internet-based asthma information, our findings may be of relevance to the management of asthma in daily life, as they suggest that experience-based beliefs about asthma triggers are shaped by prior information about asthma causality, as well as individual differences in symptom perception. The effects of prior information on generalization of trigger beliefs may be especially relevant, as they may help to explain the individual differences in asthma trigger beliefs that have been observed in individuals with asthma (Ritz et al., 2016) and associated differences in trigger avoidance strategies (Vernon et al., 2012).

### Limitations

Our findings are limited by the observation that less than half of participants responded in a consistent way to our symptom induction of 60 s inhalation of an air mixture containing 7.5% CO_2_. Although previous studies had used longer inhalation periods (ranging from 90 s to 20 min) of 7.5% CO_2_ air mixtures in order to induce respiratory symptoms or symptoms of anxiety (Bailey et al., 2005; Bogaerts et al., 2005; Pappens et al., 2012; Janssens et al., 2015), our decision to use shorter duration symptom trials was motivated by a perceived need to reduce symptom burden (participation time), and did occur after pilot testing suggesting that participants were able to differentiate between 60 s room air and CO_2_ inhalation. Nevertheless, the results of this study show that longer periods of CO_2_ inhalation may be needed to reduce variability in symptom response and increase the differences between inhalation of a 7.5% CO_2_ air mixture and room air inhalation. Furthermore, even if participants reliably responded differently to the 7.5% CO_2_ air mixture and room air, they may not have picked up on these differences in a way that would lead them to form clear symptom-trigger contingencies. In our previous experiment using 90 s inhalation of the 7.5% CO_2_ air mixture, differentiation between CS+ and CS- symptom expectancies was markedly better.

Furthermore, our findings are limited in that we did not test behavioral outcomes related to these generalized trigger beliefs, nor did we test if these generalized triggers were sensitive to disconfirmation. However, studies on fear generalization have shown that generalization of negative outcome expectancies is accompanied by increased physiological manifestations of fear, as well as increased avoidance behavior to the generalization stimuli (van Meurs et al., 2014; Dymond et al., 2015), suggesting that generalized trigger-symptom contingencies can have an impact on trigger related behaviors. Nevertheless, future studies investigating effects of extinction on generalized trigger-symptom beliefs are needed to further gauge the impact that generalization can have in this domain.

A final limitation – as in many lab-based studies – is that design decisions that were aimed at improving internal validity may have reduced external validity of our experimental design. As noted in our previous study (Janssens et al., 2015), the use of uncommon allergens as experimental asthma triggers helps to isolate specific aspects of triggers as a potential basis of trigger acquisition and generalization, but these aspects may differ from the types of potential triggers that are experienced in real life. Similarly, the selection participants that do not have a history of allergy helps us to mimic conditions that parallel an early phase of asthma trigger identification, but may preclude generalization the lived and contextualized experience of individuals with asthma that may use a variety of information to infer trigger-symptom contingencies (Caress et al., 2002; Vernon et al., 2013).

## conclusion

Our findings show that information about causality in asthma and knowledge about conceptual relationships between trigger categories influences the retention of category-based differential trigger-symptom expectancies, and generalization of these expectancies to novel trigger exemplars. Furthermore, retention and generalization of symptom expectancies was moderated by the similarity of CS+/CS- as well as similarities between CS and G categories. These findings underscore the role of higher order cognitions in contingency learning, and may help us to understand individual differences in asthma trigger beliefs that emerge over time. Finally, our findings suggest that pre-existing beliefs about asthma and asthma triggers may need to be taken into account when informing individuals with asthma about asthma trigger identification as an asthma management strategy, as these beliefs may impact subsequent learning of trigger-symptom contingencies in individuals with asthma.

## Ethics Statement

This study was carried out in accordance with the recommendations of ethical guidelines of the American Psychological Association (APA) with written informed consent from all subjects. All subjects gave written informed consent in accordance with the Declaration of Helsinki. The protocol was approved by the Medical Ethical Committee of ‘University Hospitals Leuven.’

## Author Contributions

TJ, IVD, and OVdB conceived the research questions and methodology, EC and TJ conducted the research and analyzed the data, EC, TJ, and OVdb contributed to writing the manuscript.

## Conflict of Interest Statement

The authors declare that the research was conducted in the absence of any commercial or financial relationships that could be construed as a potential conflict of interest.
