# Supplementary material for: Learning to Detect Triggers of Airway Symptoms: The Role of Illness Beliefs, Conceptual Categories and Actual Experience with Allergic Symptoms
Source: Front Psychol. 2017 Jun 7;8:926. doi: 10.3389/fpsyg.2017.00926 (PMC5461359; doi:10.3389/fpsyg.2017.00926)
Supplement: Supplementary file 1 [file Data_Sheet_1.DOCX]

Supplementary Material

Learning to detect triggers of airway symptoms: The role of illness beliefs, conceptual categories and actual experience with allergic symptoms.

**Thomas Janssens*, Eva Caris, Ilse Van Diest, Omer Van den Bergh**

*** Correspondence:** Thomas Janssens: thomas.janssens@kuleuven.be

# Supplementary Data

This supplement includes data analysis on the full set of participants, which includes participant CO_2_ response as an additional factor.

**Acquisition of trigger beliefs**

For acquisition of trigger beliefs, we constructed a multilevel model that included fixed effects of CS (CS+ vs. CS-), Trial (T1-T10), Category relationship (Similar vs. different), Trigger Information (general vulnerability vs. specific sensitivities), and CO_2_ response (responder vs. non-responder), and included all interactions between these variables.

Results are summarized in Figure 1. Overall, there was a trend for greater symptom expectancy for CS+ compared to CS- triggers, although the main effect of CS did not reach significance (F (1,912)=3.676, p=.056). However, CS effects differed based on the trigger information participants had received at the start of the experiment (CS x Trigger Information F(1,912)=4.154, p=.042), showing that participants who had received the general vulnerability information did show differential symptom expectancies to CS+ and CS- triggers(p=.004), which was not the case for participants who had received the specific sensitivities information (p=.934). This interaction was further depended upon the relationship between CS categories (F (1,912)=23.517, p<.001), showing that symptom expectancies were rated higher for CS+ exemplars compared to CS- exemplars when participants had received general vulnerability information and CS categories were more different (p<.001). This was reversed when participants had received specific sensitivity information and CS categories were more different (p=.045), For the other combinations of Trigger Information and Category Relationship, we observed no significant difference for CS+ and CS- symptom expectancies.

We also observed a main effect of trial (F(9,912)=2.385, p=.011), which was further qualified by a Trial x Trigger Information X Category Relationship (F(9,912)=1.932, p=.044) interaction. Furthermore, although we did not observe a main effect of CO_2_ response (F(1,48)=0.035, p=.853), CO_2_ Response interacted with Trial (F(9,912)=2.342, p=.013), and Trial X Trigger information (F(9,912)=2.397, p=.011), as well as with CS x Trigger Information (F(1,912)=13.752, p<.001), and CS x Category Relationship interactions (F(1,912)=14.383, p<.001). Further exploring these effects at the trial level, showed only limited consistency in CS+/CS- differentiation across trials and conditions (cf. Supplementary Figure 1). We did not observe any other significant main effects or interactions in this analysis.

**Retention of trigger beliefs and generalization to novel exemplars**

For retention of trigger beliefs, we constructed a multilevel model that included fixed effects of CS (CS+ vs. CS-), CS novelty (old vs. new), Category relationship (Similar vs. different), Trigger Information (general vulnerability vs. specific sensitivities), and CO_2_ response (responder vs. non-responder), and included all interactions between these variables. Results of this analysis is represented in Supplementary Figure 2. Overall, symptom expectancy was greater for old compared to novel trigger exemplars (main effect of CS novelty: F(1,1920)=5.048, p=.025). This effect was unmodulated by interactions with any of the other model factors. Furthermore, symptom expectancy was greater for CO_2_ non-responders compared to CO_2_ responders (main effect of CO_2_ response F(1,1920)=4.063, p=.044). We also observed an interaction of Category relationship X Trigger information (F(1,1920)=4.173, p=,041), showing that when CS categories were more different, information about specific trigger sensitivities resulted in increased symptom expectancies compared to information on general vulnerabilities (p=.014). The effect of CO_2_ response was further qualified by a CS x CO_2_ response interaction (F(1,1920)=12.646, p<.001), showing a significant difference in symptom expectancy for CS+ compared to CS- exemplars in the group of CO_2_ responders (p=.002), but not in the group of non-responders (p=.066). We also observed Trigger information X CO_2_ Response (F(1,1920)=4.442, p=.035), Category Relationship X CO_2_ response (F(1,1920)=6.244, p=.013), as well as Category relationship X Trigger information X CO_2_ response interactions (F(1,1920)=33.141, p<.001), showing that when CS categories were more different, information about specific sensitivities reduced symptom expectancies in the CO_2_ responders (p=.030), but increased symptom expectancies in the CO_2_ non-responders (p<.001). Furthermore, in the CO_2_ non-responders, information about specific sensitivities reduced symptom expectancies for participants who had received more similar CS categories during acquisition.

Finally, we observed a significant CS x Category relationship X Trigger information X CO_2_ response 4-way interaction (F(1,1920)=7.010, p=.008). Further exploration of this interaction showed that for CO_2_ responders, CS+/CS- differences only remained significant when information was given about general vulnerability and CS categories were more similar (p=.020) or when information about specific sensitivities was given and CS categories were more different (p<.001), differences between CS+/CS- for other combinations of Trigger Information and CS Category Relationship were non-significant (but in the expected direction, cf. Supplementary Figure 2a). For CO_2_ non-responders, we only observed a difference between CS+ and CS- symptom expectancies when information had been given about general vulnerability and CS categories were more similar, although this effect was not in the expected direction with symptom expectancies for CS- being greater than CS+ symptom expectancies, cf. Supplementary Figure 2b). We did not observe any other significant main effects or interactions in this analysis.

**Generalization to novel trigger categories**

Based on the trigger categories that were used as CS+ and CS-, the novel trigger categories could be coded as G+ (related to CS+), G- (related to CS-) or Gu (unrelated to both CS categories). We constructed a multilevel model that included fixed effects of Stimulus category (CS+, CS-, G+, G-, Gu), Trigger Information (general vulnerability vs. specific sensitivities), and CO_2_ response (responder vs. non-responder), and included all interactions between these variables.

Results showed a main effect of Stimulus Category (F(4,3820)=5.562, p<.001), and a main effect of CO_2_ response (F(1,3820)=20.470, p<.001). These effects were further qualified by a Stimulus category X CO_2_ response (F(4,3820)=9.943, p<.001), Trigger information x CO_2_ response (F(1,3820)=8.555, p=.003), and a Stimulus Category X Trigger Information X CO_2_ response interaction (F(4,3820)=17.084, p<.001). Further exploration of these effects showed that for CO_2_ responders, providing information about general vulnerability promoted generalization to generalization categories that were related to CS categories (G+; G-), but prevented generalization to Gu category triggers, whereas information about specific sensitivities prevented generalization to all novel categories (cf. Supplementary Figure 3a). Reverse generalization patterns were found for CO_2_ non-responders (cf. Supplementary Figure 3b). We did not observe any other significant main effects or interactions in this analysis.

# Supplementary Figures

CO_2_ Responders

*

CO_2_ Non-Responders

*

*

*

*

*

**Supplementary Figure 1**: Acquisition of differential (CS+/CS-) trigger-symptom expectancies

**Supplementary Figure 2**: Retention of trigger-symptom expectancies, depending on CS, Trigger information, and CS category Relationship for CO_2_responders (upper panel), and CO_2_ non-responders (lower panel). * CS+/CS- symptom expectancy differ at p<.05.

**Supplementary Figure 3**: Generalization of trigger-symptom contingencies according to different information groups (general vulnerability vs. specific sensitivities) for CO_2_ responders (upper panel), and CO_2_ non-responders (lower panel).
